# Sarcopenia defined by multidimensional factors and its prognostic role in heart failure: a systematic review and meta-analysis

**DOI:** 10.3389/fmed.2025.1599572

**Published:** 2025-07-21

**Authors:** Jinmei Lu, Yi Gao, Lingbo Zhou, Xinhui Peng, Haiming Feng, Zaixing Zheng

**Affiliations:** ^1^Department of Critical Care Medicine, Ningbo Medical Center Li Huili Hospital, Ningbo, Zhejiang, China; ^2^Department of Cardiology, Ningbo No. 2 Hospital, Ningbo, Zhejiang, China

**Keywords:** heart failure, sarcopenia, prognosis, meta-analysis, multi-dimensional factor

## Abstract

**Objective:**

To perform a systematic review and meta-analysis evaluating the impact of sarcopenia—defined by reductions in muscle mass, strength, and/or function—on clinical outcomes in patients with heart failure (HF), thereby informing more effective management strategies.

**Methods:**

A comprehensive literature search was conducted through 14 February 2025, using PubMed, Embase, Cochrane Library, and CNKI to identify prospective and retrospective cohort studies involving HF patients diagnosed with sarcopenia based on Asian Working Group for Sarcopenia (AWGS), European Working Group on Sarcopenia in Older People (EWGSOP2), or Ishii criteria. Data extraction was performed using standardized forms, and study quality was assessed using the Newcastle–Ottawa Scale (NOS). Meta-analytical procedures, including heterogeneity assessment and subgroup analyses, were carried out in Stata 18.0 and R 4.4.2.

**Results:**

Fifteen studies comprising 5,713 HF patients were included. Pooled analysis demonstrated that sarcopenia significantly increased the risk of adverse clinical outcomes [hazard ratio (HR) = 1.62, 95% confidence interval (CI): 1.35–1.89], including all-cause mortality (HR = 1.89, 95% CI: 1.63–2.15) and major adverse cardiovascular events (HR = 1.37, 95% CI: 1.11–1.64). Subgroup analyses revealed that sarcopenia defined by AWGS criteria and the Ishii score was significantly associated with worse outcomes (HR = 1.63, 95% CI: 1.33–1.94; HR = 1.78, 95% CI: 1.29–2.27, respectively), whereas definitions based on EWGSOP2 did not reach statistical significance (HR = 1.87, 95% CI: 0.70–3.05). Sarcopenia identified through DXA or BIA-based muscle mass assessments was also significantly correlated with adverse outcomes (DXA: HR = 1.53, 95% CI: 1.29–1.78; BIA: HR = 1.85, 95% CI: 1.10–2.61). Statistically significant associations were observed across all remaining subgroups.

**Conclusion:**

Sarcopenia, when defined using multidimensional criteria, is significantly associated with poor clinical outcomes in patients with HF. These findings underscore the importance of implementing comprehensive sarcopenia assessments to enhance prognostic evaluation and guide early intervention. Clinically, adopting multidimensional diagnostic approaches can improve risk stratification and optimize the management of HF patients.

**Systematic review registration:**

https://inplasy.com/inplasy-2025-3-0023/, identifier INPLASY202530023.

## 1 Introduction

Heart failure (HF), a clinical syndrome characterized by impaired cardiac pumping, is strongly associated with aging and manifests as a progressive decline in cardiac structure and function (1, 2). Sarcopenia, a prevalent geriatric syndrome, involves the age-related loss of skeletal muscle mass, strength, and physical function (3). These two conditions are intricately interrelated and influenced by shared risk factors, including advanced age, obesity, chronic inflammation, malnutrition, and physical inactivity (4, 5). Following the onset of HF, adaptive changes in the musculoskeletal system play a pivotal role in the manifestation of many symptoms associated with the syndrome (6).

In recent years, growing interest has emerged regarding the prognostic implications of sarcopenia in HF patients. However, findings across studies remain highly inconsistent. For example, Sato et al. (7) reported that sarcopenia—defined using the Asian Working Group for Sarcopenia (AWGS) criteria—had no significant impact on major adverse cardiovascular events (MACE) among 546 older adult HF patients aged 65 and above. In contrast, Katano et al. (8), in a study involving 534 older adult Japanese HF patients, found that AWGS-defined sarcopenia was significantly associated with all-cause mortality and served as an independent risk factor [hazard ratio (HR) = 1.65; 95% confidence interval (CI): 1.05–2.59]. Similarly, in a cohort of 272 hospitalized patients with acute HF, severe sarcopenia (AWGS-defined) independently predicted cardiac death or readmission within 1 year (HR = 2.58; 95% CI: 1.40–4.74) (9). Two studies from China examining the relationship between sarcopenia and clinical outcomes in older adult HF patients reported higher rates of readmission and MACE in sarcopenic patients compared to controls. However, no significant differences were found in endpoints such as all-cause mortality or malignant arrhythmias (10, 11). To address these conflicting findings, several meta-analyses have sought to synthesize the existing evidence. Liu et al. (12) analyzed data from 12 studies encompassing 3,696 HF patients and found that sarcopenia was significantly associated with increased risks of all-cause mortality and MACE. However, substantial heterogeneity existed due to differences in geographical populations and diagnostic definitions (12). Among the 12 studies, eight distinct sarcopenia definitions were used, with some relying solely on muscle mass. In another meta-analysis, Prokopidis et al. (13) examined 18 longitudinal studies to assess the prognostic value of sarcopenia and its components (e.g., muscle mass and grip strength) in HF. While sarcopenia defined by European Working Group on Sarcopenia in Older People (EWGSOP1) or AWGS criteria showed no significant predictive value for all-cause mortality within 1–2 years (HR = 1.35; 95% CI: 0.76–2.38), specific components—such as low psoas muscle mass at the L3–L4 level and slow gait speed—were significantly associated with increased mortality risk (13).

In recent years, growing insights into sarcopenia have led to a surge in studies investigating its impact on patients with HF. These studies increasingly adopt comprehensive and standardized diagnostic criteria, such as the AWGS (14), the revised EWGSOP2 (15), and the Ishii score-based criteria (16). In this context, we conducted a meta-analysis to examine the relationship between sarcopenia—defined using multidimensional criteria—and clinical outcomes in HF patients. This study aims to clarify the association between sarcopenia and adverse prognoses in HF and to offer clinicians evidence-based guidance for identifying high-risk patients and optimizing management strategies.

## 2 Materials and methods

### 2.1 Search strategy

Following the PRISMA guidelines (17), two reviewers independently performed a comprehensive search of PubMed, Embase, Cochrane Library, and CNKI. A hybrid retrieval strategy combining subject headings (MeSH/Emtree) and free-text terms was employed, using Boolean operators to optimize search precision. Search terms included “sarcopenia,” “heart failure,” and their synonyms. The search spanned from database inception to 14 February 2025, with no language restrictions. The full strategy is available in Supplementary Material 2. Reference lists of included studies were manually screened to identify additional relevant publications. The protocol was prospectively registered on the INPLASY platform (Registration No.: INPLASY202530023), in line with research transparency and reproducibility standards.

### 2.2 Eligibility criteria

Inclusion criteria were: (1) study design: prospective or retrospective cohort studies; (2) population: adults (≥18 years) diagnosed with HF; (3) exposure: sarcopenia diagnosed using integrated criteria involving muscle mass, strength, and function; (4) outcomes: all-cause mortality, cardiovascular mortality, or MACE (including HF rehospitalization, myocardial infarction, stroke, etc.); and (5) data: reported effect sizes [e.g., HRs and odds ratios (ORs)] with 95% CIs, along with extractable raw or adjusted data. Exclusion criteria included: (1) study type: case reports, conference abstracts, reviews, meta-analyses, animal studies, studies without control groups, or unavailable data; (2) diagnostic insufficiency: sarcopenia defined by a single parameter, such as skeletal muscle index (SMI) or grip strength alone; (3) outcome misalignment: studies reporting only intermediate biomarkers without clinical outcomes; and (4) redundancy: duplicate publications or overlapping datasets, with only the most complete report retained.

### 2.3 Data extraction and quality assessment

Data extraction adhered to the recommendations of the Cochrane Handbook for Systematic Reviews of Interventions (18). A standardized form was developed to capture key variables, including: study details (first author, year, country, and design), participant characteristics (sample size, age, and sex), sarcopenia diagnostics (criteria, instruments, thresholds for muscle mass and grip strength), and outcome indicators (e.g., all-cause mortality and MACE). Two reviewers independently extracted data, resolving discrepancies through discussion or arbitration by a third reviewer. Study quality was assessed using the Newcastle–Ottawa Scale (NOS) (19).

### 2.4 Statistical analysis

A meta-analysis was conducted using Stata 18.0 and R version 4.4.2, with all results reported in strict accordance with established methodological guidelines (17). Effect sizes—HRs or ORs with corresponding 95% CIs—were used to quantify the association between sarcopenia and clinical outcomes in patients with HF. Heterogeneity was assessed using the *Q*-test and the *I*^2^ statistic; an *I*^2^ ≥ 50% indicated substantial heterogeneity, warranting a random-effects model (DerSimonian–Laird method), while an *I*^2^ < 50% justified the use of a fixed-effects model (Mantel–Haenszel method) (18). To explore potential sources of heterogeneity, subgroup analyses were performed based on study design (prospective vs. retrospective cohorts), clinical outcomes, patient setting (inpatient vs. outpatient), muscle mass assessment method (DXA and BIA), geographic region (Asia and Europe), and sarcopenia diagnostic criteria (AWGS, EWGSOP2, and Ishii score). Sensitivity analyses were conducted by sequentially excluding individual studies to evaluate the robustness of the results. Publication bias was assessed using Egger’s test, Begg’s rank correlation test, and funnel plot analysis. Additionally, the trim-and-fill method was applied to validate the stability of the findings. A two-tailed *P* < 0.05 was considered statistically significant.

## 3 Results

### 3.1 Study selection

**FIGURE 1:**
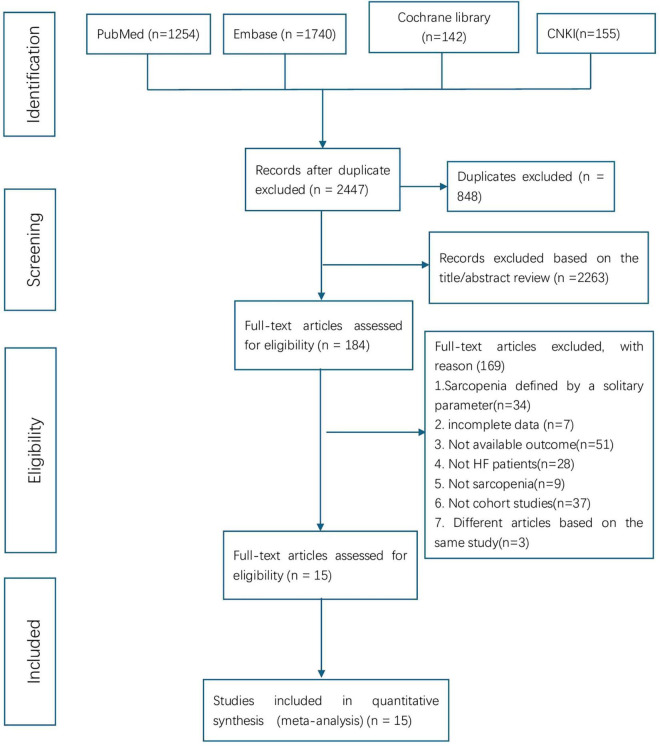
PRISMA flow chart showing the literature screening and selection process. HF, heart failure.

A systematic search of PubMed, Embase, Cochrane, and CNKI databases identified 3,291 relevant articles. After duplicate removal via EndNote, 2,447 records underwent preliminary screening. Based on predefined inclusion and exclusion criteria, 2,263 articles were excluded through title and abstract review. The remaining 184 articles underwent full-text evaluation, resulting in the exclusion of 169 studies. Ultimately, 15 high-quality studies were included in the final analysis. The screening process adhered to PRISMA guidelines and is illustrated in Figure 1.

### 3.2 Study characteristics

The final dataset comprised 15 studies involving 5,713 patients. Study characteristics and sarcopenia definitions are detailed in Tables 1, 2. Of these, eight were prospective cohort studies (8, 20–26) and seven were retrospective cohort studies (7, 9–11, 27–29). The studies included both outpatient and inpatient populations: eight from Japan, four from China, one from Türkiye, one from France, and one involving multiple European countries. All were published between 2016 and 2025. Sample sizes ranged from 119 to 1,244 participants, with a predominance of older adult patients (mean age: 70–85 years), and 3,045 (53.1%) were male. Follow-up durations ranged from 8 months to 5 years. Primary outcomes included MACE and all-cause mortality. Sarcopenia was defined using the AWGS criteria in 10 studies, the EWGSOP2 criteria in 2 studies, and the Ishii criteria in 3 studies. Notably, Onoue et al. (20) applied the Ishii score as a continuous variable in Cox regression analysis, while Kılıç et al. (28) and Maeda et al. (25) treated it as a categorical variable. To distinguish between these applications, we labeled Onoue’s method as IshiiA and the others as IshiiB.

**TABLE 1:** Characteristics of the included studies.

Study	Design	Country	Population				Follow-up duration	Outcome
			Patient	*n*	Age (year)	Gender, male (%)		
Onoue et al. (20)	Perspective	Japan	Outpatient	119	76.1 ± 6.2	73 (61%)	495 (211–715) days	MACE
Xuefeng et al. (21)	Perspective	China	Inpatient	182	Sarcopenia: 79.6 ± 5.2	Sarcopenia: 48 (64%)	36 (3–57) months	MACE
					Non-sarcopenia: 76.0 ± 4.8	Non-sarcopenia: 60 (56.1%)		
Hu et al. (22)	Perspective	China	Inpatient	182	Sarcopenia: 79.6 ± 5.2	Sarcopenia: 48 (64.0)	36 (3–57) months	MACE
					Non-sarcopenia: 76.0 ± 4.8	Non-sarcopenia: 60 (56.1)		
Nozaki et al. (23)	Perspective	Japan	Outpatient	191	73.3 ± 7.3	136 (71.2%)	8 months	MACE
Zhengguang et al. (10)	Retrospective	China	Inpatient	240	Sarcopenia: 69.5 ± 7.1	Sarcopenia: 41 (51.3%)	30.6 ± 16.7 months	MACE
					Non-sarcopenia: 70.3 ± 9.5	Non-sarcopenia: 89 (55.6%)		
Eschalier et al. (24)	Perspective	France	Inpatient	140	75.8 ± 10.2	82 (58.6%)	4 years	1. All-cause mortality
								2. MACE
Honda et al. (9)	Retrospective	Japan	Inpatient	272	Sarcopenia: 78 (70–85)	Sarcopenia: 29 (63.0)	1 year	All-cause mortality
					Non-sarcopenia: 76 (65–82)	Non-sarcopenia: 141 (62.4)		
Xiaolin et al. (11)	Retrospective	China	Inpatient	300	Sarcopenia: 76.3 ± 12.2	Sarcopenia: 52 (51.0)	45.1 ± 3.1 months	MACE
					Non-sarcopenia: 76.6 ± 11.3	Non-sarcopenia: 108 (54.5)		
Formiga et al. (27)	Retrospective	Europe	Outpatient	226	80.0 ± 5.0	103 (45.6)	2 years	All-cause mortality
Kılıç et al. (28)	Retrospective	Türkiye	Outpatient	722	70.1 ± 8.4	306 (42.4)	2 years	1. All-cause mortality
								2. Hospitalization
Saito et al. (26)	Perspective	Japan	Inpatient	269	Sarcopenia: 85 (78–87)	Sarcopenia: 88 (65.7)	690 (459–730) days	All-cause mortality
					Non-sarcopenia: 80 (73–86)	Non-sarcopenia: 47 (34.8)		
Sato et al. (7)	Retrospective	Japan	Inpatient	546	77 (72–82)	309 (56.6)	0.90 (0.35–1.89) years	MACE
Shakuta et al. (29)	Retrospective	Japan	Inpatient	546	70 (58–78)	347 (63.6)	5 years	All-cause mortality
Maeda et al. (25)	Perspective	Japan	Inpatient	1,244	Sarcopenia: 77 (71–83)	Sarcopenia: 360 (53.3)	1 year	All-cause mortality
					Non-sarcopenia: 84 (80–88)	Non-sarcopenia: 363 (61.8)		
Katano et al. (8)	Perspective	Japan	Inpatient	534	Sarcopenia: 79.1 ± 7.2	Sarcopenia: 90 (32%)	2.04 ± 1.06 years	All-cause mortality
					Non-sarcopenia: 78.8 ± 7.3	Non-sarcopenia: 65 (30%)		

MACE, Major Adverse Cardiovascular Events.

**TABLE 2:** Diagnostic criteria and cutoff value used to define sarcopenia in meta-analysis.

Study	Diagnostic criteria						
	Definition	Low muscle mass		Low muscle strength		Low physical performance	
		Cutoff value	Measure	Cutoff value	Measure	Cutoff value	Measure
Onoue et al. (20)	IshiiA	Sarcopenia score: men: 0.62 × (age − 64) − 3.09 × (grip strength − 50) − 4.64 × (calf circumference − 42); women: 0.80 × (age − 64) − 5.09 × (grip strength − 34) − 3.28 × (calf circumference − 42); man: sarcopenia score ≥ 105; woman: sarcopenia score ≥ 120					
Xuefeng et al. (21)	AWGS	Man SMI < 7.0 kg/m^2^	DXA	Man < 26 kg	HG	<0.8 m/s	GS
		Woman SMI < 5.4 kg/m^2^		Woman < 18 kg			
Hu et al. (22)	AWGS	Man SMI < 7.0 kg/m^2^	DXA	Man < 26 kg	HG	<0.8 m/s	GS
		Woman SMI < 5.4 kg/m^2^		Woman < 18 kg			
Nozaki et al. (23)	AWGS	Man SMI < 7.0 kg/m^2^	BIA	Man < 26 kg	HG	<0.8 m/s	GS
		Woman SMI < 5.7 kg/m^2^		Woman < 18 kg			
Zhengguang et al. (10)	AWGS	NR	DXA	Man < 25 kg	HG	<0.8 m/s	GS
				Woman < 18 kg			
Eschalier et al. (24)	EWGSOP2	Man SMI < 10.75 kg/m^2^	BIA	Man < 30 kg	HG	<0.8 m/s	GS
		Woman SMI < 6.75 kg/m^2^		Woman < 20 kg			
Honda et al. (9)	AWGS	Man SMI < 7.0 kg/m^2^	BIA	Man < 28 kg	HG	<1.0 m/s	GS
		Woman SMI < 5.7 kg/m^2^		Woman < 18 kg			
Xiaolin et al. (11)	AWGS	Man SMI < 7.0 kg/m^2^	DXA	Man < 28 kg	HG	<1.0 m/s	GS
		Woman SMI < 5.4 kg/m^2^		Woman < 18 kg			
Formiga et al. (27)	EWGSOP2	Man ASM < 20 kg	BIA	Man < 27 kg	HG	SPPB ≤ 8	
		Woman ASM < 15 kg		Woman < 16 kg			
Kılıç et al. (28)	IshiiB	Sarcopenia score: men: 0.62 × (age − 64) − 3.09 × (grip strength − 50) − 4.64 × (calf circumference − 42); women: 0.80 × (age − 64) − 5.09 × (grip strength − 34) − 3.28 × (calf circumference − 42); man: sarcopenia score ≥ 105; woman: sarcopenia score ≥ 120					
Saito et al. (26)	AWGS	Man SMI < 7.0 kg/m^2^	DXA	Man < 28 kg	HG	GS < 1.0 m/s, five-time chair-stand test ≥ 12 s, or SPPB ≤ 9.	
		Woman SMI < 5.4 kg/m^2^		Woman < 18 kg			
Sato et al. (7)	AWGS	Man SMI < 7.0 kg/m^2^	BIA	Man < 28 kg	HG	GS < 1.0 m/s, five-time chair-stand test ≥ 12 s, or SPPB ≤ 9.	
		Woman SMI < 5.7 kg/m^2^		Woman < 18 kg			
Shakuta et al. (29)	AWGS	Man SMI < 7.0 kg/m^2^	Predictive	Man < 28 kg	HG	GS < 1.0 m/s, five-time chair-stand test ≥ 12 s, or SPPB ≤ 9.	
		Woman SMI < 5.4 kg/m^2^	Equation	Woman < 18 kg			
Maeda et al. (25)	IshiiB	Sarcopenia score: men: 0.62 × (age − 64) − 3.09 × (grip strength − 50) − 4.64 × (calf circumference − 42); women: 0.80 × (age − 64) − 5.09 × (grip strength − 34) − 3.28 × (calf circumference − 42); man: sarcopenia score ≥ 141; woman: sarcopenia score ≥ 165					
Katano et al. (8)	AWGS	Man SMI < 7.0 kg/m^2^	DXA	Man < 28 kg	HG	GS < 1.0 m/s, five-time chair-stand test ≥ 12 s, or SPPB ≤ 9.	
		Woman SMI < 5.4 kg/m^2^		Woman < 18 kg			

AWGS, Asian Working Group for Sarcopenia; DXA, dual X-ray absorptiometry; SMI, Skeletal Muscle Mass Index; HG, handgrip; GS, gait speed; BIA, bioelectrical impedance analysis; NR, not reported; ASM, Appendicular Skeletal Muscle Mass; SPPB, Short Physical Performance Battery.

### 3.3 Quality assessment

Study quality was systematically assessed using the NOS, with results summarized in Table 3. The average score was 7.4. Thirteen studies (86.7%) were classified as high quality (≥7 points), while two (13.3%) were of moderate quality (<7 points). All studies clearly specified inclusion and exclusion criteria (e.g., age, HF phenotype, and absence of baseline outcome events). Sarcopenia and non-sarcopenia groups were derived from the same cohorts and assessed using objective diagnostic tools. All studies adjusted for key confounders using multivariate models (e.g., Cox regression). Twelve studies (80%) used medical records or standardized follow-up data to evaluate clinical outcomes. While 13 studies (86.7%) had a median follow-up of over 1 year, only 6 (40%) reported loss-to-follow-up rates, which ranged from 1.6% to 8.8%.

**TABLE 3:** Quality assessment of included articles.

	Selection					Outcome			
Study	Representativeness of the exposed cohort	Selection of the non-exposed cohort	Ascertainment of exposure	Demonstration that outcome of interest was not present at start of study	Comparability	Assessment of outcome	Was follow-up long enough for outcomes to occur	Adequacy of follow-up of cohorts	Total points
Onoue et al. (20)	1	1	1	1	1	1	1	0	7
Xuefeng et al. (21)	1	1	1	1	2	0	1	1	8
Hu et al. (22)	1	1	1	1	1	0	1	1	7
Nozaki et al. (23)	1	1	1	1	1	1	0	0	6
Zhengguang et al. (10)	1	1	1	1	2	1	1	1	9
Eschalier et al. (24)	1	1	1	1	2	0	1	0	7
Honda et al. (9)	1	1	1	1	1	1	1	1	8
Xiaolin et al. (11)	1	1	1	1	1	1	1	0	7
Formiga et al. (27)	1	1	1	1	2	1	1	0	8
Kılıç et al. (28)	0.5	1	1	1	1.5	1	1	0	7
Saito et al. (26)	1	1	1	1	2	1	1	0	8
Sato et al. (7)	1	1	1	1	1	1	0	1	7
Shakuta et al. (29)	0.5	1	0.5	1	1.5	1	1	0	6.5
Maeda et al. (25)	1	1	1	1	1	1	1	0	7
Katano et al. (8)	1	1	1	1	2	1	1	0	8

### 3.4 Results of the meta-analysis

**FIGURE 2:**
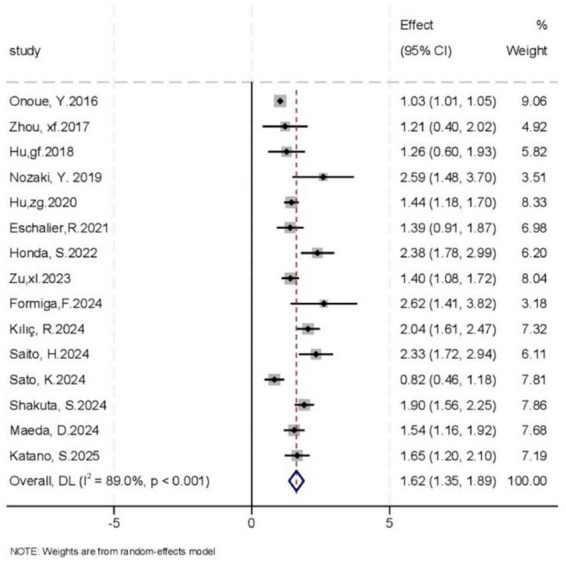
Forest plot including all studies on adverse clinical outcomes.

**FIGURE 3:**
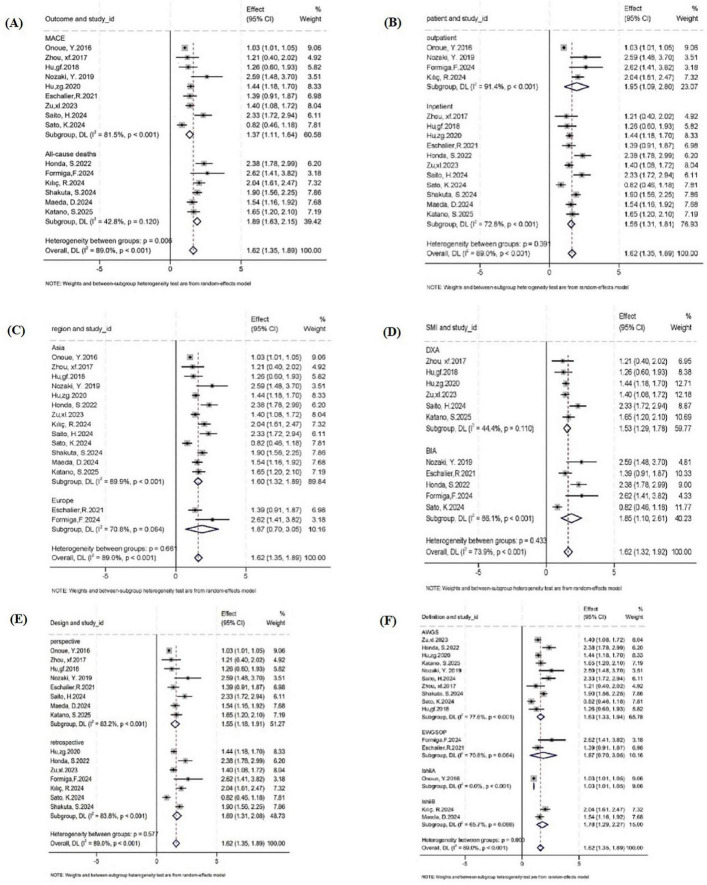
Forest plot of adverse clinical outcomes. Subgroup analyses by **(A)** clinical outcomes, **(B)** patient types, **(C)** region, **(D)** muscle mass assessment, **(E)** study types, and **(F)** sarcopenia diagnostic criteria. AWGS, Asian Working Group for Sarcopenia; EWGSOP, European Working Group on Sarcopenia in Older People; DXA, dual X-ray absorptiometry; BIA, bioelectrical impedance analysis.

Meta-analysis results demonstrated that sarcopenia significantly increased the risk of adverse clinical outcomes in patients with HF (HR = 1.62, 95% CI: 1.35–1.89; *I*^2^ = 89.0%). The corresponding forest plot is presented in Figure 2, with subgroup analysis plots shown in Figure 3. Subgroup analyses based on predefined variables revealed differential associations between sarcopenia and adverse outcomes across various contexts. Specifically, sarcopenia significantly increased the risks of both all-cause mortality (HR = 1.89, 95% CI: 1.63–2.15; *I*^2^ = 42.8%) and MACE (HR = 1.37, 95% CI: 1.11–1.64; *I*^2^ = 81.5%). With respect to diagnostic criteria, sarcopenia defined by AWGS (HR = 1.63, 95% CI: 1.33–1.94; *I*^2^ = 77.6%) and the Ishii score (HR = 1.78, 95% CI: 1.29–2.27; *I*^2^ = 65.7%) was associated with increased risk, whereas the EWGSOP2-based definition (HR = 1.87, 95% CI: 0.70–3.05; *I*^2^ = 70.8%) did not yield a statistically significant association. Regarding muscle mass assessments, sarcopenia diagnosed by DXA (HR = 1.53, 95% CI: 1.29–1.78; *I*^2^ = 44.4%) or BIA (HR = 1.85, 95% CI: 1.10–2.61; *I*^2^ = 86.1%) was significantly associated with adverse outcomes. Stratified by study design, sarcopenia was linked to poor outcomes in both prospective (HR = 1.55, 95% CI: 1.18–1.91; *I*^2^ = 83.2%) and retrospective cohort studies (HR = 1.69, 95% CI: 1.31–2.08; *I*^2^ = 83.8%). Similarly, sarcopenia was predictive of adverse outcomes in both outpatient (HR = 1.95, 95% CI: 1.09–2.80; *I*^2^ = 91.4%) and inpatient populations (HR = 1.62, 95% CI: 1.31–1.81; *I*^2^ = 72.8%).

### 3.5 Sensitivity analysis and publication bias

**FIGURE 4:**
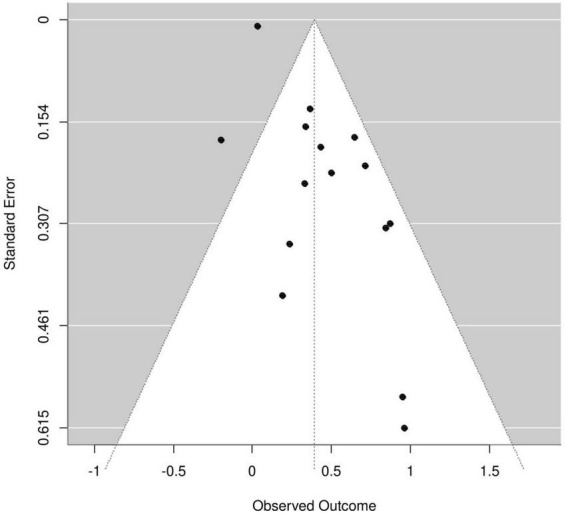
Funnel plots of the 15 included studies.

**FIGURE 5:**
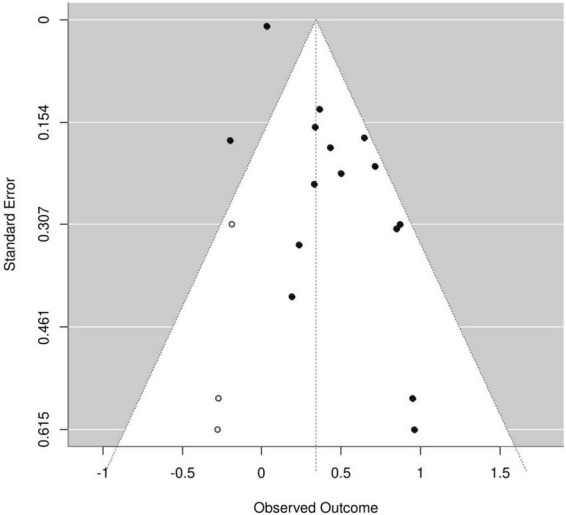
Funnel plot of 15 studies and 3 virtual-studies after trim and fill correction.

An asymmetric funnel plot (Figure 4) was observed. While Egger’s test indicated significant publication bias (*P* < 0.001), Begg’s rank correlation test did not (*P* = 1.0), suggesting potential bias. Using the trim-and-fill method to add three hypothetical studies, the adjusted pooled HR was 1.41 (95% CI: 1.19–1.67), indicating a slight attenuation but retained significance—demonstrating the robustness of the original findings. The corrected funnel plot (Figure 5) showed improved symmetry. Sensitivity analysis, conducted by sequential exclusion of individual studies, confirmed the stability of the association between sarcopenia and adverse HF outcomes.

## 4 Discussion

This analysis aimed to provide evidence-based guidance for clinical risk stratification and management in this population. Our meta-analysis included 15 cohort studies with a total of 5,713 participants. The results demonstrated that sarcopenia, when defined using multidimensional diagnostic criteria, is an independent risk factor for poor prognosis in HF patients (HR = 1.62; 95% CI: 1.35–1.89), corroborating findings from prior studies. For instance, among 534 older adult HF patients, AWGS-defined sarcopenia was significantly associated with all-cause mortality (HR = 1.65) (8). Similarly, Liu et al.’s (12) meta-analysis of 12 studies with 3,696 patients found increased risks of all-cause death and MACE in sarcopenic individuals. Our findings suggest that multidimensionally defined sarcopenia is closely associated with adverse clinical outcomes in HF, highlighting the complex interplay between the two conditions. They share multiple risk factors—such as aging, obesity, chronic inflammation, malnutrition, and sedentary behavior—and the musculoskeletal adaptations that follow HF onset significantly contribute to symptom burden (4, 6, 30). Subgroup analyses further revealed that sarcopenia defined using AWGS or the Ishii score was significantly associated with adverse outcomes in HF patients. In contrast, sarcopenia defined by EWGSOP2 criteria did not show a statistically significant association. However, this result should be interpreted cautiously. Only two studies in our analysis used the EWGSOP2 criteria, with a combined sample size of just 366. The limited data and small sample size may have reduced the statistical power and reliability of this finding. Future research with larger, well-designed cohorts is warranted to clarify the prognostic relevance of EWGSOP2-defined sarcopenia in HF.

In our meta-analysis, 80% of the included studies (12/15) did not stratify HF subtypes, with a notable absence of investigations specifically targeting HF with reduced ejection fraction (HFrEF). Only three studies addressed distinct HF phenotypes: (1) Xiaolin et al. (11) identified sarcopenia as an independent risk factor for adverse cardiovascular events in 300 patients with HF with mildly reduced ejection fraction (HFmrEF); (2) Kılıç et al. (28) demonstrated that sarcopenia was associated with increased hospitalization and mortality in 722 HFmrEF patients; and (3) Zhengguang et al. (10) reported an independent association between sarcopenia and adverse outcomes (HR = 1.439) in 240 patients with HF with preserved ejection fraction (HFpEF). Exploratory analysis of HFmrEF cohorts revealed a significant association between sarcopenia and prognosis (OR = 1.70, 95% CI: 1.08–2.33; Supplementary Figure 1); however, the substantial heterogeneity (*I*^2^ = 81.7%) warrants cautious interpretation. Given the distinct pathophysiological mechanisms underlying HF subtypes—where neurohormonal activation predominates in HFrEF and metabolic dysregulation is more characteristic of HFpEF—the complete lack of HFrEF-specific data represents a critical gap. Future research should prioritize the evaluation of sarcopenia’s prognostic relevance across all HF phenotypes, particularly in HFrEF populations.

Our study employed a multidimensional diagnostic framework for sarcopenia, integrating assessments of muscle mass, strength, and physical performance. This approach reflects the dual structural and functional pathology of sarcopenia, characterized by loss of muscle mass and reduced muscle function. Sole reliance on muscle mass indicators—such as SMI measured via computed tomography (CT)—risks overlooking the broader metabolic impairments affecting muscle tissue. Conversely, isolated functional metrics like grip strength cannot fully capture variations in nutritional status or physical reserves across individuals (15). The dual-aspect assessment adopted in our study aligns with the multidimensional diagnostic criteria endorsed by both the EWGSOP and the AWGS (14, 15). Traditional single-parameter definitions are limited in their ability to reflect the complex pathophysiological profile of sarcopenia (31). In contrast, a combined diagnostic model enhances precision by identifying individuals who truly meet the criteria for sarcopenia, thereby reducing the risk of misclassification (32). In this study, sarcopenia defined using both the AWGS and Ishii score criteria was significantly associated with adverse HF outcomes, suggesting that a multidimensional approach more effectively captures prognosis-related determinants. While each diagnostic framework has unique strengths, AWGS and EWGSOP2—though more operationally complex—are well-suited for comprehensive inpatient screening due to their thorough inclusion of functional and structural indicators. In contrast, the Ishii score offers a practical, rapid screening method more appropriate for outpatient settings (16, 20). By leveraging these complementary tools according to the clinical context, diagnostic accuracy can be optimized.

The Ishii score is a simple, cost-effective screening tool for sarcopenia, integrating three readily obtainable parameters: age, grip strength, and calf circumference. It facilitates efficient risk stratification in primary care and low-resource settings by circumventing the need for advanced imaging modalities such as CT, MRI, or DXA (16). Prior studies have confirmed its high sensitivity and specificity in identifying sarcopenia among community-dwelling older adults. The original cut-off values (≥105 for men, ≥120 for women) demonstrated robust predictive performance in the initial validation cohort (20). However, in specific populations such as HF patients, tailored cut-off values may yield greater diagnostic precision. For instance, a study of 1,244 older adult HF patients found that adjusted thresholds (≥141 for men, ≥165 for women) provided superior prognostic and diagnostic accuracy (25). Despite underlying heterogeneity, our subgroup analysis indicated that sarcopenia defined by the Ishii score was significantly associated with poor HF prognosis (HR = 1.78), underscoring its utility in risk prediction. In summary, the Ishii score is a practical and economical option for initial sarcopenia screening, particularly in outpatient and resource-limited settings. However, to enhance its clinical utility, diagnostic thresholds should be calibrated to specific patient populations. Future multicenter studies are warranted to establish HF-specific Ishii scoring systems and define dynamic intervention thresholds.

This study has several multidimensional limitations. First, there is substantial geographic bias: 80% of the 15 included studies were conducted in Asian populations (Japan: 8; China: 4), while only 3 involved European cohorts, and none represented African or Latin American populations. This geographic imbalance limits the generalizability of the findings. Second, follow-up durations were skewed, with 93.3% (14/15) of studies reporting follow-up periods of ≤4 years (including two studies with <1 year), and only one study exceeding 5 years, thereby limiting insights into the ultra-long-term (≥5 years) prognostic impact of sarcopenia. Third, HF subtype stratification was insufficient, as most studies failed to differentiate between HFrEF and HFpEF, introducing uncontrolled heterogeneity into subgroup analyses and leaving a critical evidence gap for HFrEF. Fourth, the risk of residual confounding is high: key lifestyle factors—such as protein intake and moderate-to-vigorous physical activity—were not consistently adjusted for, despite their bidirectional relationships with sarcopenia, complicating causal inference. Fifth, methodological heterogeneity was evident. Several single-center studies had small sample sizes (<300 patients), and inconsistent diagnostic criteria for sarcopenia (e.g., varying DXA/BIA cutoffs) contributed to result variability. Finally, publication bias cannot be ruled out, as the potential underreporting of negative results may have led to an overestimation of sarcopenia’s effect magnitude.

## 5 Conclusion

Sarcopenia, when assessed using multidimensional criteria, is significantly associated with adverse clinical outcomes in patients with HF. Such a comprehensive approach offers improved prognostic precision and opportunities for timely intervention. Clinically, adopting a multidimensional evaluation strategy for sarcopenia and implementing early targeted interventions may enhance patient outcomes.

## Data Availability

The original contributions presented in this study are included in this article and Supplementary Material 3 (Dataset for Analysis). Further inquiries can be directed to the corresponding author.
