# Supplementary material for: Sarcopenia defined by multidimensional factors and its prognostic role in heart failure: a systematic review and meta-analysis
Source: Front Med (Lausanne). 2025 Jul 21;12:1599572. doi: 10.3389/fmed.2025.1599572 (PMC12319046; doi:10.3389/fmed.2025.1599572)
Supplement: Supplementary file 1 [file Image_1.pdf]

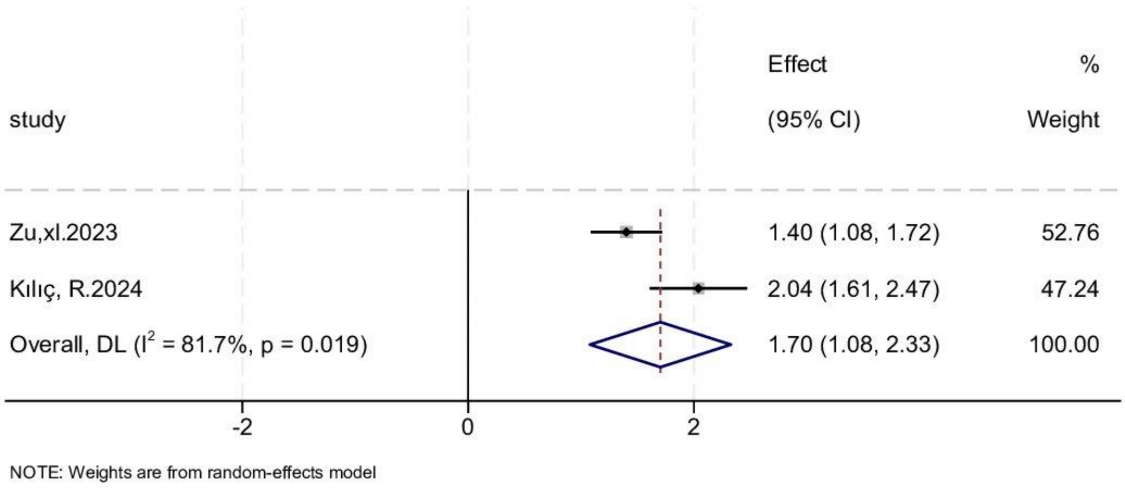

Supplementary Figure 1.

Forest plot of sarcopenia association with adverse clinical outcomes in HFmrEF. HFmrEF, Heart Failure with Mid-Range Ejection Fraction.
