# Supplementary material for: Sarcopenia defined by multidimensional factors and its prognostic role in heart failure: a systematic review and meta-analysis
Source: Front Med (Lausanne). 2025 Jul 21;12:1599572. doi: 10.3389/fmed.2025.1599572 (PMC12319046; doi:10.3389/fmed.2025.1599572)
Supplement: Supplementary file 3 [file Table_2.DOCX]

| **Search Strategies（Search Date: 2025.2.14）** | | |
| --- | --- | --- |
| **Pubmed** | | |
| Search | Query | Results |
| #1 | "Sarcopenia"[Mesh] OR sarcopenia OR "muscle atrophy" OR "muscle wasting" OR "muscle loss" OR "muscular atrophy" | 58755 |
| #2 | "Heart Failure"[Mesh] OR "heart failure" OR "congestive heart failure" OR "cardiac failure" OR "heart insufficiency" | 296639 |
| #3 | #1 and #2 | 1254 |
| **EMBASE** | | |
| #1 | sarcopenia:ti,ab,kw OR 'muscle atrophy':ti,ab,kw OR 'muscle wasting':ti,ab,kw OR 'muscle loss':ti,ab,kw OR 'muscular atrophy':ti,ab,kw OR 'sarcopenia quality of life questionnaire' | 70581 |
| #2 | ('heart failure':ti,ab,kw OR 'congestive heart failure':ti,ab,kw OR 'cardiac failure':ti,ab,kw OR 'heart insufficiency':ti,ab,kw OR exp) AND 'heart failure' | 417758 |
| #3 | #1 AND #2 | 1744 |
| **Cochrane** | | |
| #1 | MeSH descriptor: [Sarcopenia] explode all trees | 991 |
| #2 | sarcopenia OR "atrophy of muscle" OR "muscle wasting" OR "muscle loss" OR "muscular atrophy" | 4809 |
| #3 | #1 or #2 | 4809 |
| #4 | MeSH descriptor: [Heart Failure] explode all trees | 15032 |
| #5 | “heart failure” OR “congestive heart failure” OR “cardiac failure” OR “heart insufficiency” | 41274 |
| #6 | #4 or #5 | 41304 |
| #7 | #3 and #6 | 142 |
| **Chinese National Knowledge Infrastructure(CNKI)** | | |
| #1 | 主题 = （肌少症 OR 肌肉减少症 OR 肌肉萎缩 OR 肌肉量减少 OR 肌肉功能减退） | 18,548 |
| #2 | 主题 = （心力衰竭 OR 充血性心力衰竭 OR 心脏衰竭 OR 心功能不全） | 174,724 |
| #3 | #1 and #2 | 155 |
