# Supplementary material for: Sarcopenia defined by multidimensional factors and its prognostic role in heart failure: a systematic review and meta-analysis
Source: Front Med (Lausanne). 2025 Jul 21;12:1599572. doi: 10.3389/fmed.2025.1599572 (PMC12319046; doi:10.3389/fmed.2025.1599572)
Supplement: Supplementary file 5 [file Table_4.DOCX]

**PRISMA-S Checklist**

| **Section/topic** | **#** | **Checklist item** | **Location(s) Reported** |
| --- | --- | --- | --- |
| **INFORMATION SOURCES AND METHODS** | | | |
| Database name | 1 | Name each individual database searched, stating the platform for each. | PubMed (NCBI), EMBASE (Elsevier), Cochrane Library (Wiley), CNKI (China National Knowledge Infrastructure) |
| Multi-database searching | 2 | If databases were searched simultaneously on a single platform, state the name of the platform, listing all of the databases searched. | Not applied (searches conducted individually in each database) |
| Study registries | 3 | List any study registries searched. | No study registries (e.g., ClinicalTrials.gov) were searched |
| Online resources and browsing | 4 | Describe any online or print source purposefully searched or browsed (e.g., tables of contents, print conference proceedings, web sites), and how this was done. | No online resources (e.g., society websites, conference proceedings) were browsed |
| Citation searching | 5 | Indicate whether cited references or citing references were examined, and describe any methods used for locating cited/citing references (e.g., browsing reference lists, using a citation index, setting up email alerts for references citing included studies). | Reference lists of included studies were manually screened to identify additional relevant publications. This method was used alongside database searches detailed in the document. |
| Contacts | 6 | Indicate whether additional studies or data were sought by contacting authors, experts, manufacturers, or others. | No authors or experts were contacted for additional data |
| Other methods | 7 | Describe any additional information sources or search methods used. | No other search methods (e.g., handsearching books or grey literature) were used |
| **SEARCH STRATEGIES** | | | |
| Full search strategies | 8 | Include the search strategies for each database and information source, copied and pasted exactly as run. | The full search strategy is available in Supplementary Material 2. |
| Limits and restrictions | 9 | Specify that no limits were used, or describe any limits or restrictions applied to a search (e.g., date or time period, language, study design) and provide justification for their use. | No limits or restrictions (e.g., date range, language, study design) were applied |
| Search filters | 10 | Indicate whether published search filters were used (as originally designed or modified), and if so, cite the filter(s) used. | No published search filters (e.g., Cochrane RCT filters) were used. |
| Prior work | 11 | Indicate when search strategies from other literature reviews were adapted or reused for a substantive part or all of the search, citing the previous review(s). | No adaptation or reuse of search strategies from other literature reviews was performed. |
| Updates | 12 | Report the methods used to update the search(es) (e.g., rerunning searches, email alerts). | No search updates were conducted. |
| Dates of searches | 13 | For each search strategy, provide the date when the last search occurred. | The last search for all databases (PubMed, Embase, Cochrane Library, CNKI) occurred on February 14, 2025. |
| **PEER REVIEW** | | | |
| Peer review | 14 | Describe any search peer review process. | No specific search peer review process was mentioned. |
| **MANAGING RECORDS** | | | |
| Total Records | 15 | Document the total number of records identified from each database and other information sources. | Records identified from each database:  - PubMed: 1254 records were retrieved.  - Embase: 1744 records were obtained.  - Cochrane Library: 142 records were found.  - CNKI: 155 records were identified.  No records were obtained from other information sources |
| Deduplication | 16 | Describe the processes and any software used to deduplicate records from multiple database searches and other information sources. | To deduplicate records from multiple database searches, EndNote software was employed. First, all identified records (1254 from PubMed, 1744 from Embase, 142 from Cochrane Library, and 155 from CNKI, totaling 3,291 relevant articles) were imported into EndNote. The software then automatically detected and flagged duplicate records by matching criteria such as title, author names, journal title, publication year, and volume - issue - page information. After the automated check, a manual review was also conducted to confirm the removal of duplicates and ensure no false - positive or false - negative identifications. Through this process, duplicates were eliminated, and 2,447 unique records moved forward for preliminary screening. This deduplication procedure ensured the integrity of the subsequent screening and selection stages, aligning with best practices for systematic review conduct. |
|  |  |  |  |
| PRISMA-S: An Extension to the PRISMA Statement for Reporting Literature Searches in Systematic Reviews | | |  |
| Rethlefsen ML, Kirtley S, Waffenschmidt S, Ayala AP, Moher D, Page MJ, Koffel JB, PRISMA-S Group. | | |  |
| Last updated February 27, 2020. | |  |  |
